# Effect of Cleaning Multiple-Funnel Traps on Captures of Bark and Woodboring Beetles in Northeastern United States

**DOI:** 10.3390/insects11100702

**Published:** 2020-10-14

**Authors:** Kevin J. Dodds, Marc F. DiGirolomo

**Affiliations:** U.S. Forest Service, Forest Health Protection, Region 9 State and Private Forestry, 271 Mast Road, Durham, NH 03824, USA; Marc.F.DiGirolomo@usda.gov

**Keywords:** Teflon, surfactant, survey, trapping, trap maintenance

## Abstract

**Simple Summary:**

Semiochemical-baited traps are used to survey insect communities and detect invasive species. These traps are left in the field during the growing season where large amounts of pollen and other debris can build up on smooth trapping surfaces. There was a concern this buildup would provide an escape route for some insects and interfere with trapping results. We tested the effects of this pollen buildup on captures of bark and woodboring beetles in northeastern forests in two experiments. While many beetles did not respond to treatments, we found a positive effect of trap cleaning for three bark beetles and one cerambycid species. The response of other species was more nuanced.

**Abstract:**

Two experiments were conducted in mixed hardwood-conifer forests in the northeastern United States to test the effects of cleaning surfactant and non-surfactant treated multiple-funnel traps used to catch bark and woodboring beetles. Large amounts of pollen and other debris often form a crust on the interior of traps (personal observations). Such surface deposits may provide footholds for beetles to escape capture in traps. In one experiment, we tested cleaned surfactant and non-surfactant traps against non-cleaned surfactant and non-surfactant traps. In a second experiment, we tested field cleaning of modified multiple-funnel traps as an alternative to substituting clean traps on each collection visit. There was no effect of surfactant treated traps, cleaned or not, on total beetles or individual bark beetle species captured. However, in situ cleaned traps were statistically better at capturing total beetles, total bark beetles, and several bark beetle species than non-cleaned control traps. Surfactant-treated non-modified traps and cleaned modified traps had higher species richness and abundance than other treatments at the site level. Our results suggest that cleaning traps to remove accumulated pollen and debris may be helpful for some species but would have limited benefit for broad-scale trapping of bark and woodboring beetles in northeastern forests.

## 1. Introduction

Semiochemical-baited traps targeting bark and woodboring beetles are ubiquitous tools used in exotic species surveys, population monitoring efforts, and scientific investigations. These traps can be used to investigate native communities and survey for potentially damaging exotic species [1,2,3]. Large-scale national surveys, such as USDA Forest Service Early Detection and Rapid Response and USDA Animal and Plant Health Inspection Service Cooperative Agricultural Pest Survey, rely on these traps as an important tool to screen for potentially damaging insects at high risk sites throughout the U.S. [4,5]. Semiochemical-baited traps offer a simpler, more cost-effective technique to sample insect communities than visual surveys, active sampling, or plant material rearing.

While the use of semiochemicals to survey wood-inhabiting insects has expanded over the past two decades, the effects of various factors that could influence trap success are unknown. These factors can be broken into two categories: (1) those related to traps themselves (intrinsic), and (2) those related to factors outside of the traps (extrinsic). Important intrinsic factors include trap types [6,7,8,9], lure placement on the trap [10], preservation and collection method [11,12], and surface treatments [13,14]. Extrinsic factors that can influence trapping include habitat selection [15,16], vertical and horizontal placement in a habitat [8,17,18,19], site disturbance [1], and local wind and weather patterns [20,21].

Surface treatments such as the application of a surfactant solution to trap surfaces can greatly increase the abundance of woodborers captured in multiple-funnel and intercept panel traps [13,14]. Keeping trap surfaces clean from pollen buildup and other material may also increase trap catches. During previous trapping efforts, we have noted large amounts of pollen on the inside and outside of funnels of multiple-funnel traps, especially from traps placed in pine forests. Pollen, along with dust and other debris, builds up on the traps and hardens as the summer progresses creating a noticeable rough texture on both surfactant-treated traps and non-treated traps. This increased texture may facilitate trap escape by insects or counter the beneficial effects of surfactant treatments.

The influence of surface cleaning treatments on the efficacy of traps for bark beetles (Coleoptera: Curculionidae: Scolytinae) and woodboring beetles (Coleoptera: Cerambycidae and Buprestidae) was tested in two experiments established in northeastern mixed hardwood-conifer forests in the northeastern United States. These experiments tested the response of bark and woodboring beetles to traps with and without surfactants that were either substituted with clean traps or cleaned in the field at the time of trap collections.

## 2. Materials and Methods

### 2.1. Experiment 1: Effect of Pollen/Dirt Accumulation on Surfactant-Treated and Non-Treated Traps on Trapping Efficacy

To test the effects of pollen and other accumulation on bark beetle and woodborer trap catches, an experiment was established on a forested site managed by the University of New Hampshire in Durham, New Hampshire (43.1486° N, 70.9358° W). The area had a large opening that had been expanded through periodic cutting over the previous five years that resulted in a mix of downed wood decay classes and stumps. The surrounding forest was a mix of *Pinus strobus* L., *Pinus resinosa* Ait., *Tsuga canadensis* (L.) Carr., *Picea abies* (L.) Karst, *Acer rubrum* L., and *Betula lenta* L.

**Figure 1:**
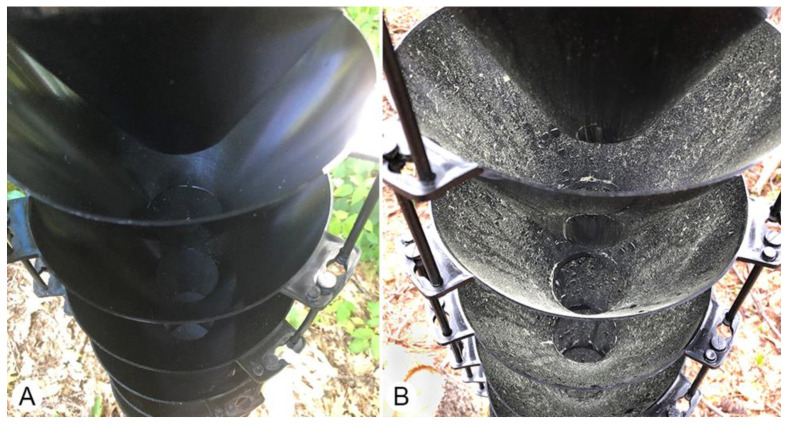
Example of multiple-funnel trap treatments from Experiment 1 in a mixed conifer stand in southeastern New Hampshire. (**A**) Clean trap without surfactant switched out biweekly with a clean trap; (**B**) Control trap without surfactant after six weeks in the field (not shown: two additional treatments of surfactant-treated clean trap and surfactant-treated control trap).

Four trap treatments were established using standard 12-unit multiple-funnel [22] traps: (1) non-surfactant treated control trap that was left in situ for the duration of the experiment, (2) clean non-surfactant treated trap that was replaced biweekly, (3) surfactant-treated control trap left in situ for the duration of the experiment, and (4) clean surfactant-treated trap that was replaced biweekly (Figure 1). Teflon Non-stick Dry-film Lubricant (Dupont, Wilmington, DE, USA) was used for surfactant treatments and applied to the inside and outside of each funnel in treated funnel traps once at the beginning of the experiment. During biweekly insect collections, the clean non-surfactant traps and clean surfactant-treated traps were swapped with identical traps that had been cleaned with light soap and water. The clean surfactant-treated traps were not retreated with Teflon when replaced. All traps were baited with ultra-high release α-pinene pouch lure (~2 g/day), ultra-high release ethanol pouch lure (~0.4 g/day), ipsenol bubblecap lure (0.4 mg/d), and monochamol bubblecap lure (0.8 mg/d). All lures were purchased from Synergy Semiochemicals, Delta, BC. Traps were hung from a piece of conduit with a 90° bend placed upright into the ground. Collecting cups were approximately 0.5–1.0 m above the ground. Propylene glycol antifreeze solution (Prestone^®^ RV Waterline, Lake Forest, IL, USA) was used as a collection and preservation liquid in collection cups. Seven replicate blocks containing each of the four treatments were established along the forested edge of the clearing. Treatments were separated by ≥10 m and blocks were ≥30 m apart. The experiment was established 16 May 2019and ended 22 August 2019, with lures changed at the midpoint.

### 2.2. Experiment 2: Field Cleaning Traps-Effect of Pollen/Dirt Accumulation on Trap Efficacy

To further test the effects of trap cleaning on bark beetle and woodborer captures, an experiment was established on the Massabesic Experimental Forest (43.4491° N, 70.6584° W) near Alfred, Maine. The area used for trapping was a mix of *P. resinosa*, *P. strobus*, *Quercus rubra* L., *A. rubrum*, and other hardwoods. A wildlife opening (~0.5 ha) had been created the previous winter and all downed trees were cut into 2–4 m pieces and stacked in large piles.

**Figure 2:**
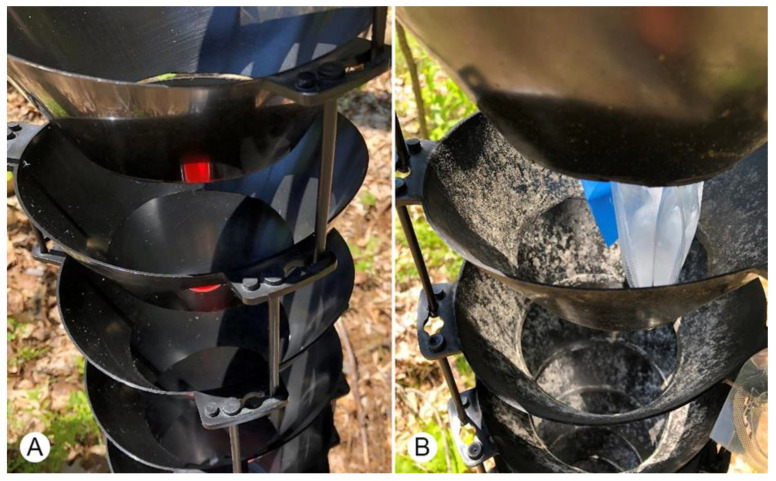
Example of modified multiple-funnel trap treatments from Experiment 2 after six weeks in a mixed hardwood-pine stand in southern Maine. (**A**) Trap cleaned with baby wipes during biweekly collections. (**B**) Control trap that was clean at the beginning of the season and then never maintained.

Two trap treatments were established at the site using modified [10] 12-unit multiple-funnel traps that had enlarged funnel holes to allow semiochemicals to sit inside traps: (1) non-treated control traps and (2) in-situ treatment traps cleaned biweekly. Traps were not treated with Teflon. Treatment traps were hand cleaned with non-alcohol baby wipes (Huggies Natural Care, Kimberly Clark Corporation, Neenah, WI, USA). Multiple wipes were used on each trap to thoroughly clean around the internal and external portions of each funnel (Figure 2). Less than five minutes was spent cleaning each treatment trap. Traps were then left to air dry. Each trap was baited with ultra-high release α-pinene pouch lure, ultra-high release ethanol pouch lure, ipsenol bubble cap lure, and monochamol bubble cap lure (Synergy Semiochemicals). Release rates for lures were identical to those described for Experiment 1. Traps were hung from cord tied between two trees, with traps at least 3 m from each tree and the collecting cup at least 0.5 m above the ground. Propylene glycol antifreeze solution (Prestone^®^ RV Waterline, Lake Forest, IL, USA) was used as a collection and preservation liquid. Seven replicates of the two treatments were established on the forested edge of the wildlife opening. Treatments were separated by ≥10 m and blocks were ≥30 m apart. The experiment was established on 5 June 2019 and ended 23 August 2019, with lures changed at the midpoint of the experiment.

### 2.3. Sample Collection and Processing

All insect collections were poured through paint strainers in the field, labeled, and sealed in zip-top bags that were returned to the laboratory and frozen until further processing. Samples were later thawed, sorted by family, and identified to species using appropriate taxonomic resources [23,24,25,26]. The cerambycids *Monochamus carolinensis* (Olivier) and *Monochamus titillator* (F.) were combined as *Monochamus* complex because these species are often difficult to separate using morphological characteristics. Voucher specimens were deposited in the U.S. Forest Service, Durham Field Office Forest Insect Collection (DFOC), Durham, NH, USA.

### 2.4. Statistics

Data preparation and analyses were similar for both experiments. Trap collections were pooled by trap across the entire trapping season and species that represented ≥1% of total beetles collected were statistically analyzed. Comparisons among treatments were analyzed using a generalized linear mixed model (PROC GLIMMIX, version 9.3; SAS Institute, Cary, NC, USA) via maximum likelihood estimation with replicates as blocks. Replicates were random factors in each experiment, with trap treatments as fixed effects. The negative binomial function with log link was used to model data. When needed, Tukey’s honestly significant difference test was used to separate differences among trap treatments.

Community level estimates, including species richness, abundance, Simpson’s 1-D, and Chao-1 were calculated using pooled data by trap type for each experiment. Individual-based rarefaction curves were also used to compare species richness among treatments. All community level estimates and rarefaction curves were conducted using PAST [27].

## 3. Results

### 3.1. Experiment 1: Effect of Pollen/Dirt Accumulation on Surfactant-Treated and Non-Treated Traps on Trapping Efficacy

A total of 32,192 beetles from 103 species were captured during the experiment (Table S1). Cerambycids accounted for 12.8% of the beetles and 39 species, while bark beetles accounted for 87% of the total beetles and 50 species. Buprestids accounted for only 0.2% of the total beetles and 14 species. The most common cerambycids were *Monochamus scutellatus* (Say) (6.7% of total beetles) and *Asemum striatum* (L.) (2.9%). Some bark beetle species were captured in relatively large numbers, including *Dryocoetes autographus* (Ratz.) (18.1%), *Ips grandicollis* (Eichhoff) (16.8%), *Orthotomicus caelatus* (Eichhoff) (16.1%), *Dendroctonus valens* LeConte (10.3%), *Crypturgus pusillus* (Gyllenhal) (9.0%), *Gnathotrichus materiarius* (Fitch) (5.5%), and *Hylastes porculus* (Erichson) (4.0%). *Buprestis consularis* Gory was the most common buprestid accounting for 0.04% of total beetle catches. Beetles captured five times or less accounted for 44% of the species, while 21.4% of species were singletons. Ten species of Scolytinae captured represent new state records for New Hampshire: *Crypturgus alutaceus* Schwarz, *Crypturgus borealis* Swaine, *Heteroborips seriatus* (Blandford), *Hypothenemus californicus* Hopkins, *Hypothenemus dissimilis* (Zimmermann), *Phloeotribus piceae* Swaine, *Pityophthorus lautus* Eichhoff, *Pseudopityophthorus asperulus* (LeConte), *Xyleborus affinis* Eichhoff, and *Xyleborus intrusus* Blandford.

**Figure 3:**
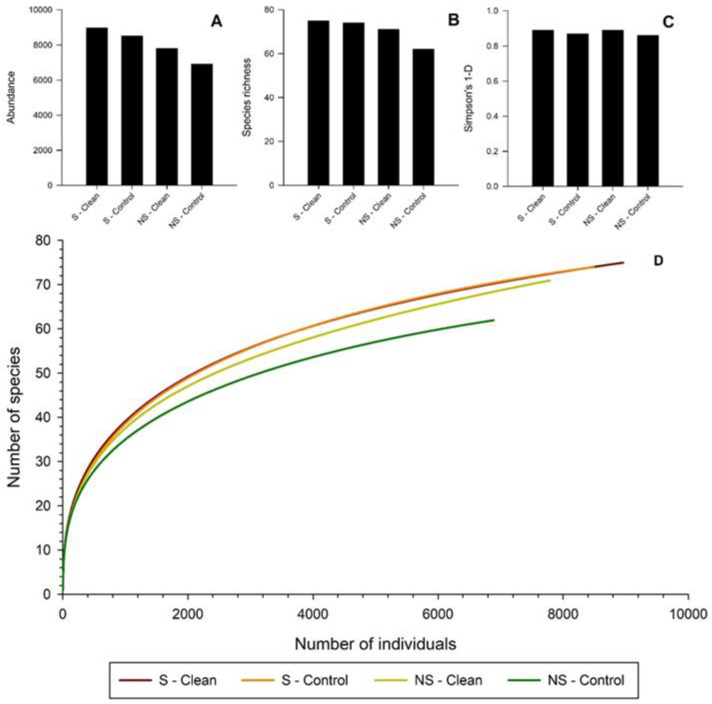
Abundance and diversity metrics for beetles captured in clean vs. control multiple-funnel traps with (S) and without surfactant (NS) treatment during Experiment 1 in southeastern New Hampshire. (**A**) Abundance of bark and woodboring beetles; (**B**) Species richness; (**C**) Simpson’s 1-D estimates; (**D**) Individual-based rarefaction curves.

Abundance and species richness were higher in surfactant treated traps than non-surfactant treated traps (Figure 3A,B). However, average species richness did not vary among the treatments (F_3,18_ = 1.28, *p* = 0.3). Simpson’s 1-D estimates were similar among all treatments, with clean surfactant treated traps only slightly higher than other treatments (Figure 3C). Chao-1 estimates were highest for non-surfactant clean traps (92) and surfactant cleaned traps (90), followed by surfactant control (82) and non-surfactant control traps (73). Individual-based rarefaction curves were nearly identical within the surfactant treated traps (Figure 3D). Non-surfactant control traps had the lowest species richness according to the rarefaction curves.

There were no significant differences among treatments for total beetles captured (F_3,18_ = 1.28, *p* = 0.3) or total bark beetles captured (F_3,18_ = 0.6, *p* = 0.6) (Table 1). However, surfactant treated clean and control traps captured more total cerambycids than the non-surfactant traps (F_3,18_ = 12.6, *p* = 0.0001, Table 1). The cerambycid *A. striatum* was captured in the largest numbers in the surfactant treated control traps (F_3,18_ = 4.6, *p* = 0.01; Table 1). Surfactant treated control traps captured significantly more *A. striatum* than non-surfactant treated cleaned traps but were similar to surfactant cleaned traps and non-surfactant treated control traps. *Monochamus scutellatus* was captured in significantly higher numbers in surfactant treated cleaned traps compared to non-surfactant cleaned traps (F_3,18_ = 6.2, *p* = 0.005; Table 1). Surfactant treated cleaned traps were similar to surfactant treated control and non-surfactant treated control traps. There were no significant differences among the treatments for any bark beetle tested, including *C. alutaceus* (F_3,18_ = 2.7, *p* = 0.08), *C. pusillus* (F_3,18_ = 2.9, *p* = 0.06), *D. valens* (F_3,18_ = 1.8, *p* = 0.2), *D. autographus* (F_3,18_ = 2.8, *p* = 0.07), *G. materiarius* (F_3,18_ = 0.14, *p* = 0.9), *Hylastes opacus* (Erichson) (F_3,18_ = 0.8, *p* = 0.5), *H. porculus* (F_3,18_ = 1.3, *p* = 0.3), *I. grandicollis*, (F_3,18_ = 0.9, *p* = 0.5) and *O. caelatus* (F_3,18_ = 2.0, *p* = 0.2) (Table 1).

### 3.2. Experiment 2: Field Cleaning Traps-Effect of Pollen/Dirt Accumulation on Trap Efficacy

A total of 6734 beetles from 88 species were captured during the experiment (Table S2). Forty-two cerambycid species were identified and accounted for 44.3% of total beetles captured. Bark beetles accounted for 55.6% of total beetles captured and 35 species. Buprestids were relatively rare and only accounted for 0.3% of total beetles and 11 species. The most common cerambycids captured included *M. scutellatus* (23.6%), *A. striatum* (10.4%), *Monochamus* complex (2.73%) and *Monochamus notatus* (Drury) (1.8%). Dominant bark beetles included *I. grandicollis* (20.4%), *D. autographus* (13.1%), *O. caelatus* (4.9%), *D. valens* (3.9%), *G. materiarius* (3.6%), and *H. porculus* (3.5%). Buprestids were rare, with *Dicerca divaricata* (Say) being the most common and only accounting for 0.1% of total beetle catches. The majority of species (59.1%) were captured five times or less, with 31.8% captured only one time. Three species of Scolytinae represent new state records for Maine: *Crypturgus alutaceus* Schwarz, *Hypothenemus californicus* Hopkins, and *Micracis suturalis* LeConte.

**Figure 4:**
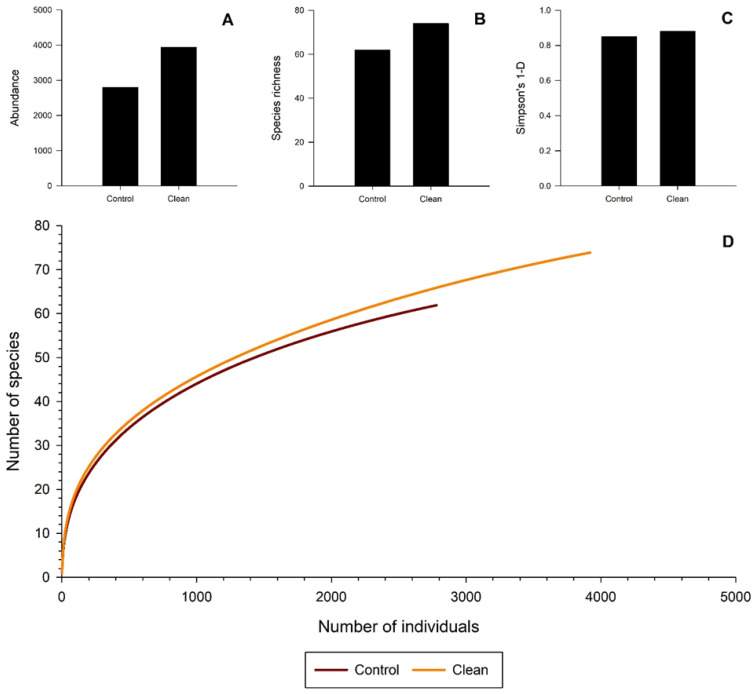
Abundance and diversity metrics for beetles captured in clean vs. control modified multiple-funnel traps during Experiment 2 in southern Maine. (**A**) Abundance of bark and woodboring beetles captured; (**B**) Species richness; (**C**) Simpson’s 1-D estimates; (**D**) Individual-based rarefaction curves.

Total abundance, species richness, and species diversity were higher in cleaned than control traps (Figure 4A–C). Average species richness was similar between control and clean traps (F_1,6_ = 4.4, *p* = 0.08; Table 2). Chao-1 estimates suggested that more species were available to sample, with estimates of 76 and 91 species for control and cleaned traps, respectively. Rarefaction also suggested that cleaned traps captured more species than control traps (Figure 4D).

Total number of beetles captured were significantly higher in cleaned than control traps (F_1,6_ = 6.8, *p* = 0.04; Table 2). This was also the case for bark beetles (F_1,6_ = 16.5, *p* = 0.007; Table 2). There was no difference in captures between cleaned and control traps for cerambycids (F_1,6_ = 0.6, *p* = 0.5; Table 2). Twelve beetle species met the minimum threshold of 1% total captures to be statistically analyzed and the majority of these showed no effect of trap cleaning. Cleaned traps did not capture significantly more *A. striatum* (F_1,6_ = 1.9, *p* = 0.2), *M. scutellatus* (F_1,6_ = 0.01, *p* = 0.9), *Monochamus* complex (F_1,6_ = 0.04, *p* = 0.9), *M. notatus* (F_1,6_ = 0.9, *p* = 0.4), *D. autographus* (F_1,6_ = 2.3, *p* = 0.2), *H. porculus* (F_1,6_ = 4.0, *p* = 0.09), *I. grandicollis* (F_1,6_ = 2.8, *p* = 0.15), or *G. materiarius* (F_1,6_ = 5.4, *p* = 0.06) (Table 2). Significantly higher catches in cleaned traps were found for *D. valens* (F_1,6_ = 11.3, *p* = 0.02), *O. caelatus* (F_1,6_ = 15.6, *p* = 0.008), *Pityophthorus puberulus* (LeConte) (F_1,6_ = 22.2, *p* = 0.003), and *Acanthocinus obsoletus* (Olivier) (F_1,6_ = 10.1, *p* = 0.02) (Table 2).

## 4. Discussion

A common tactic to improve trap efficacy for bark beetle and woodborer surveys is to apply a surface treatment to increase trap slickness (reduce surface friction) causing insects to fall more readily through the trap funnels into the collection cup. Keeping traps clean from buildup of pollen and other substances encountered during use may serve a similar purpose, increasing slickness and reducing footholds for alighting insects, and resulting in higher trap catches. However, our two experiments conducted to test surfactant treatments and trap cleaning in northeastern U.S. forests using standard and modified multiple-funnel traps generally failed to show any benefit for most species of bark and woodboring beetles, and only marginal improvements when considering community level sampling such as species richness.

There was no response to surfactant treated traps, cleaned or not, in terms of average total beetles captured in Experiment 1. However, in-situ cleaned traps in Experiment 2 were statistically better at capturing average total beetles than non-cleaned control traps. Surfactant-treated traps in Experiment 1 and cleaned traps in Experiment 2 all contained higher species richness and higher abundance than other treatments or controls at the site level. Rarefaction curves also demonstrated that surfactant treated traps in Experiment 1 and cleaned traps in Experiment 2 had higher species richness. No curves reached an asymptote suggesting sampling was incomplete in both experiments for all treatments.

The positive effects of intercept trap surfactant treatments on cerambycid captures is well documented [12,13,14,28], whereas results from the current experiments were mixed. While more total cerambycids were captured in either of the surfactant treated traps compared to the non-surfactant traps in Experiment 1, this pattern did not hold for individual species. Only two species were abundant enough to statistically analyze (*A. striatum* and *M. scutellatus*), however, and whereas the average catches from surfactant treatments were always higher than or equal to some treatments, the surfactant treated traps did not always statistically separate from non-surfactant treated traps. A similar observation was previously made for *Tetropium* spp. in panel traps treated with a surfactant [29]. *Monochamus*, a genus that has previously shown a strong response to surfactants on traps [12,28], had similar catches across clean surfactant treated, control surfactant treated and control non-surfactant treated traps. Clean surfactant treated traps were the only treatment with greater abundance than clean non-surfactant treated traps, suggesting no benefit of trap cleaning for *Monochamus*. Further, within surfactant and non-surfactant treated traps, cleaning traps never increased catches. Similarly, in Experiment 2, very little effect of pollen and grime removal on trap catches was noted for cerambycids even though the look and feel of these traps were very different (see Figure 2). Only *A. obsoletus* was captured in statistically higher numbers in cleaned traps in Experiment 2. This species has also been captured in higher numbers in surfactant treated traps [28]. No response was noted between the trap treatments for total cerambycids, *A. striatum*, *M. scutellatus*, *Monochamus* complex, and *M. notatus*, suggesting trap cleaning is unnecessary for most cerambycids. Unfortunately, low captures of buprestids did not allow for comparison among treatments in either experiment.

Surfactants have not been tested as thoroughly for bark beetles as they have for cerambycids and other woodborers [12,13,30]. There was no effect of surfactant or trap cleaning for total bark beetles or individual bark beetle species that were statistically compared during Experiment 1. Results from Experiment 2 were more nuanced and suggested an overall trend toward higher catches in cleaned traps, but with only a few comparisons statistically significant (e.g., total bark beetles, *D. valens*, *O. caelatus*, and *P. puberulus*). Previous experiments conducted in Louisiana have also indicated little effect of multiple-funnel surface treatments on bark beetle captures [28]. *Hylastes porculus* and *I. grandicollis* were captured in Louisiana and during the current experiments, with no effect of surfactant or trap cleaning found for either species. Turpentine beetles were also captured in Louisiana and the northeast, with *Dendroctonus terebrans* (Olivier) in the southeast and *D. valens* in the northeast. These species occupy a similar niche and are behaviorally similar. No response to surfactants was noted in either region for either species. However, *D. valens* was captured in higher numbers in cleaned traps during Experiment 2, suggesting if this species is a target of trapping efforts, trap cleaning may be beneficial.

Two different trap types were used in the current experiments. In Experiment 1, standard multiple-funnel traps were used, while modified multiple-funnel traps with enlarged funnel holes [10] were used in Experiment 2. In limited comparisons, modified multiple-funnel traps are as good as or better than standard multiple-funnel traps for catching bark beetles and woodborers [7,10]. Standard multiple-funnel traps coated with surfactants were more efficient at capturing woodborers than untreated traps [14]. Modified multiple-funnel traps have not been tested with surfactants. We found only a small benefit of trap treatment for cerambycids, and no response for bark beetles, when using standard multiple-funnel traps. Modified multiple-funnel traps in Experiment 2 demonstrated some benefit for bark beetles but only limited benefit for cerambycids when traps were cleaned. It is possible that enlarging the openings of funnels in multiple-funnel traps has the same net effect of using surfactants on standard traps.

Two different treatments, surfactant treatment and surface cleaning, were used to make the trap more slippery for arriving insects. Previous research has demonstrated the usefulness of surfactant treatments [12,13,14,28]. However, less is known about potential benefits of trap cleaning. Certainly, visual and tactile observations made during the study clearly suggested that cleaned traps were more slippery than dirty traps that were covered in hardened pollen, pine resin, dirt, and grime. This roughened surface would suggest possible footholds and escape routes for incoming insects. However, only a very limited response to removal of this debris was found in Experiment 1, with a more mixed response documented in Experiment 2. Part of the confusion in the results could be due to different levels of debris that accumulated on traps at the sites or the manner in which traps were cleaned. Pollen and debris appeared to have accumulated to a lesser degree on traps in Experiment 1 where very few differences were noted compared to traps in Experiment 2 where more differences were found. Tree species composition, stand openness, wind patterns, moisture, and topography may all contribute to varying degrees in how quickly a trap accumulates pollen and debris. A relationship between the amount and type of debris building on traps and trap efficacy could exist, but more study would be needed to determine this. Further, it is possible that our trap cleaning treatments had unexpected consequences. Cleaning the funnel surfaces may have removed some of the surfactant in Experiment 1, likely resulting in a less effective surfactant treatment as the trapping season progressed. The baby wipes we used in Experiment 2 contained fewer ingredients than many other brands, but it is unknown how any residual components might influence insects around traps, acting as either an attractant, repellant, or by removing the obvious grime but merely spreading around the finer debris. Finally, flight phenology of individual species may also influence results, with species dispersing early in the season encountering less differences among trap treatments than those dispersing later. However, we noted no seasonal pattern within the results.

## 5. Conclusions

Various factors can help improve trap efficacy when surveying bark beetles and woodborers [9,10,11,14,15,18]. Surfactant treatments are an intrinsic factor that are occasionally employed in bark beetle and woodborer surveys to increase trapping results. We hypothesized that trap cleaning may have a similar effect on trap catches and be a suitable substitute for surfactant treatments than can be difficult to apply and costly. However, cleaning traps, either by replacing dirty traps with clean traps or in-situ cleaning with baby wipes had only limited benefit during bark beetle and woodborer trapping in northeastern forests. Cleaning traps to remove accumulated pollen and debris may be helpful for some species, but it is generally not necessary to improve collections.

## Figures and Tables

**Table 1:** Mean (±SE) total beetles, species richness, total bark beetles, total cerambycids, and individual species that accounted for ≥1% of total beetles captured in trap treatments during Experiment 1. Means followed by the same letter are not significantly different (*p* > 0.05).

Species	Surfactant Treated		No Surfactant Treatment		*p*-Value
	Clean	Control	Clean	Control	
Total beetles	1217.2 ± 196.3	1113.4 ± 179.6	1020.5 ± 164.7	965.5 ± 155.8	0.2151
Species richness	38.5 ± 2.7	38.8 ± 2.7	35.5 ± 2.6	33. 4± 2.5	0.3
Total bark beetles	1018.0 ± 177.1	934.3 ± 162.5	919.9 ± 160.0	852.2 ± 148.3	0.6013
*Crypturgus alutaceus*	18.4 ± 6.7	9.8 ± 3.7	11.6 ± 4.3	9.2 ± 3.5	0.08
*Crypturgus pusillus*	139.7 ± 44.3	67.4 ± 21.5	111.4 ± 35.4	51.8 ± 16.6	0.06
*Dendroctonus valens*	111.6 ± 45.3	77.9 ± 31.7	75.9 ± 30.9	65.5 ± 26.7	0.2
*Dryocoetes autographus*	155.9 ± 29.9	218.2 ± 41.6	155.1 ± 29.7	248.9 ± 47.4	0.07
*Gnathotrichus materiarius*	56.3 ± 10.8	59.6 ± 11.4	60.6 ± 11.6	55.4 ± 10.6	0.9
*Hylastes opacus*	19.1 ± 4.3	13.7 ± 3.2	19.0 ± 4.3	16.0 ± 3.6	0.51
*Hylastes porculus*	30.6 ± 19.1	16.6 ± 10.5	24.3 ± 15.2	13.8 ± 8.7	0.3
*Ips grandicollis*	174.5 ± 24.3	219.8 ± 30.4	187.6 ± 26.1	173.5 ± 24.1	0.5
*Orthotomicus caelatus*	205.4 ± 52.6	171.2 ± 43.9	140.7 ± 36.1	127.9 ± 32.9	0.2
Total Cerambycidae	189.8 ± 23.5a	180.2 ± 22.3a	92.2 ± 11.7b	114.5 ± 14.4b	0.0001
*Asemum striatum*	32.1 ± 6.6ab	47.5 ± 9.6a	20.6 ± 4.4b	26.8 ± 5.6ab	0.01
*Monochamus scutellatus*	108.6 ± 19.5a	84.6 ± 15.3ab	45.2 ± 8.4b	58.4 ± 10.7ab	0.005

**Table 2:** Mean (±SE) total beetles, species richness, total bark beetles, total cerambycids, and individual species that accounted for ≥1% of total beetles captured in trap treatments during Experiment 2.

Species	Control	Cleaned	*p*-Value
Total beetles	395.7 ± 41.1	556.7 ± 57.5	0.04
Species richness	29.2 ± 2.4	35.6 ± 2.7	0.08
Total bark beetles	191.7 ± 23.7	329.1 ± 40.2	0.007
*Dryocoetes autographus*	52.4 ± 8.4	73.7 ± 11.6	0.2
*Dendroctonus valens*	10.6 ± 2.2	25.9 ± 4.9	0.02
*Hylastes porculus*	10.3 ± 3.2	17.3 ± 5.2	0.09
*Ips grandicollis*	77.1 ± 14.3	108.7 ± 20.0	0.1
*Gnathotrichus materiarius*	12.6 ± 2.4	21.0 ± 3.7	0.06
*Orthotomicus caelatus*	13.3 ± 2.6	32.9 ± 5.8	0.008
*Pityophthorus puberulus*	2.9 ± 0.8	12.6 ± 2.5	0.003
Total cerambycids	201.1 ± 20.6	225.0 ± 23.0	0.5
*Acanthocinus obsoletus*	2.0 ± 0.7	6.9 ± 1.9	0.02
*Asemum striatum*	44.0 ± 5.6	56.1 ± 7.0	0.2
*Monochamus scutellatus*	110.3 ± 14.5	111.8 ± 14.7	0.9
*Monochamus* complex	12.6 ± 4.1	13.7 ± 4.4	0.9
*Monochamus notatus*	9.2 ± 2.3	7.2 ± 1.8	0.4

## Supplementary Materials

The following are available online at https://www.mdpi.com/2075-4450/11/10/702/s1, Table S1: Species list and abundance of bark beetles and woodborers captured during Experiment 1, Table S2: Species list and abundance of bark beetles and woodborers captured during Experiment 2.
