# Correction: Dodds, K.J.; DiGirolomo, M.F. Effect of Cleaning Multiple-Funnel Traps on Captures of Bark and Woodboring Beetles in Northeastern United States. *Insects* 2020, *11*, 702

**DOI:** 10.3390/insects12020103

**Published:** 2021-01-25

**Authors:** Kevin J. Dodds, Marc F. DiGirolomo

**Affiliations:** U.S. Forest Service, Forest Health Protection, Region 9 State and Private Forestry, 271 Mast Road, Durham, NH 03824, USA; Marc.F.DiGirolomo@usda.gov

The authors wish to make the following corrections to this paper [1]:

On pages 5 and 6, we reported that 13 new state records of Scolytinae, 10 in New Hampshire and 3 in Maine, were collected during these experiments.

It was brought to our attention that five of these species were previously reported in [2] and, therefore, do not represent new state records. Consequently, we wish to change the following text:

1. On page 5, change the last sentence of paragraph one: “Ten species of Scolytinae captured represent new state records for New Hampshire: *Crypturgus alutaceus* Schwarz, *Crypturgus borealis* Swaine, *Heteroborips seriatus* (Blandford), *Hypothenemus californicus* Hopkins, *Hypothenemus dissimilis* (Zimmermann), *Phloeotribus piceae* Swaine, *Pityophthorus lautus* Eichhoff, *Pseudopityophthorus asperulus* (LeConte), *Xyleborus affinis* Eichhoff, and *Xyleborus intrusus* Blandford” to: “Six species of Scolytinae captured represent new state records for New Hampshire: *Crypturgus alutaceus* Schwarz, *Heteroborips seriatus* (Blandford), *Hypothenemus californicus* Hopkins, *Hypothenemus dissimilis* (Zimmermann), *Pityophthorus lautus* Eichhoff, and *Xyleborus intrusus* Blandford.”
2. On page 6, change the last sentence of paragraph two: “Three species of Scolytinae represent new state records for Maine: *Crypturgus alutaceus* Schwarz, *Hypothenemus californicus* Hopkins, and *Micracis suturalis* LeConte.” to: “Two species of Scolytinae represent new state records for Maine: *Crypturgus alutaceus* Schwarz and *Hypothenemus californicus* Hopkins.”

The authors would like to apologize for any inconvenience caused to the readers by these changes.
