# Supplementary material for: Effect of Cleaning Multiple-Funnel Traps on Captures of Bark and Woodboring Beetles in Northeastern United States
Source: Insects. 2020 Oct 14;11(10):702. doi: 10.3390/insects11100702 (PMC7602418; doi:10.3390/insects11100702)
Supplement: Supplementary file 1 [file insects-11-00702-s001.pdf]

Table S1. Species list and abundance of bark beetles and woodborers captured during Experiment 1.

**Scolytinae**		**Cerambycidae**		**Buprestidae**	
Species	Total	Species	Total	Species	Total
Ambrosiophilus atratus (Eichhoff)	95	*Acanthocinus obsoletus* (Olivier)	62	*Agrilus anxius* Gory	1
Anisandrus sayi Hopkins	3	*Acanthocinus pusillus* Kirby	36	*Agrilus obsoletoguttatus* Gory	1
Conophthorus coniperda (Schwarz)	7	*Acmaeops p. proteus* (Kirby)	40	*Buprestis consularis* Gory	12
Corthylus columbianus Hopkins	1	*Anelaphus parallelus* (Newman)	14	*Buprestis maculativentris* Say	2
Cryphalus ruficollis Hopkins	12	*Anoplodera pubera* (Say)	3	*Buprestis maculipennis* Gory	1
Crypturgus alutaceus Schwarz	466	*Asemum australe* LeConte	6	*Buprestis striata* F.	9
Crypturgus borealis Swaine	25	*Asemum striatum* (L.)	948	*Chalcophora virginiensis* (Drury)	1
Crypturgus pusillus (Gyllenhal)	2885	*Astylopsis sexguttata* (Say)	10	*Chrysobothris dentipes* (Germar)	8
Cyclorhipidion pelliculosum (Eichhoff)	1	*Bellamira scalaris* (Say)	1	*Chrysobothris femorata* (Olivier)	1
Dendroctonus valens LeConte	3316	*Brachyleptura brevis* (Kirby)	6	*Chrysobothris harrisi* Hentz	3
Dryocoetes affaber (Mannerheim)	32	*Brachyleptura circumdata* (Olivier)	2	*Chrysobothris scabripennis* Gory & Laporte	7
Dryocoetes autographus (Ratzeburg)	5816	*Clytus marginicollis* Laporte & Gory	3	*Chrysobothris trinervia* Kirby	1
Euwallacea validus (Eichhoff)	39	*Clytus ruricola* (Olivier)	18	*Dicerca divaricata* (Say)	5
Gnathotrichus materiarius (Fitch)	1761	*Cyrtophorus verrucosus* (Olivier)	7	*Phaenops fulvoguttata* (Harris)	1
Heteroborips seriatus (Blandford)	2	*Etorofus subhamatus* (Randall)	1		
Hylastes opacus Erichson	518	*Eupogonius tomentosus* (Haldeman)	1		
Hylastes porculus Erichson	1295	*Evodinus monticola* (Randall)	6		
Hylesinus aculeatus Say	30	*Graphisurus fasciatus* (Degeer)	1		
Hylesinus criddlei (Swaine)	1	*Judolia cordifera* (Olivier)	57		
Hylurgopinus rufipes (Eichhoff)	1	*Lepturges confluens* (Haldeman)	1		
Hylurgops pinifex (Fitch)	136	*Lepturopsis biforis* (Newman)	2		
Hypothenemus californicus Hopkins	2	*Monochamus notatus* (Drury)	63		
Hypothenemus dissimilis (Zimmermann)	3	*Monochamus scutellatus* (Say)	2142		
Ips grandicollis (Eichhoff)	5406	*Monochamus* spp. *(carolinensis* or *titillator)*	42		
Ips pini (Say)	11	*Neoclytus acuminatus* (F.)	116		
Lymantor decipiens (LeConte)	1	*Orthosoma brunneum* (Forster)	15		
Monarthrum fasciatum (Say)	8	*Prionus laticollis* (Drury)	1		

Monarthrum mali (Fitch)	37	*Rhagium i. inquisitor* (L.)	295
Orthotomicus caelatus (Eichhoff)	5180	*Stictoleptura c. canadensis* (Olivier)	11
Orthotomicus latidens (LeConte)	2	*Strangalepta abbreviata* (Germar)	2
Phloeotribus liminaris (Harris)	1	*Strophiona nitens* (Forster)	1
Phloeotribus piceae Swaine	1	*Tetropium cinnamopterum* Kirby	4
Pityogenes hopkinsi Swaine	292	*Tetropium schwarzianum* Casey	41
Pityokteines sparsus (LeConte)	5	*Trachysida mutabilis* (Newman)	3
Pityophthorus cariniceps LeConte	8	*Trigonarthris subpubescens* (Kirby)	2
Pityophthorus consimilis LeConte	6	*Typocerus v. velutinus* (Olivier)	2
Pityophthorus lautus Eichhoff	2	*Xylotrechus colonus* (F.)	8
Pityophthorus puberulus (LeConte)	165	*Xylotrechus integer* (Haldeman)	143
Pityophthorus sp.	2	*Xylotrechus s. sagittatus* (Germar)	19
Polygraphus rufipennis (Kirby)	7		
Pseudopityophthorus asperulus (LeConte)	3		
Pseudopityophthorus minutissimus (Zimmermann)	8		
Trypodendron lineatum (Olivier)	1		
Xyleborinus attenuatus (Blandford)	43		
Xyleborinus saxesenii (Ratzeburg)	34		
Xyleborus affinis Eichhoff	2		
Xyleborus intrusus Blandford	2		
Xyleborus xylographus (Say)	26		
Xylosandrus germanus (Blandford)	277		
Xyloterinus politus (Say)	27		

Table S2. Species list and abundance of bark beetles and woodborers captured during Experiment 2.

**Scolytinae**		**Cerambycidae**		**Buprestidae**	
Species	Total	Species	Total	Species	Total
Anisandrus sayi Hopkins	1	*Acanthocinus obsoletus* (Olivier)	67	*Buprestis striata* F.	1
Crypturgus alutaceus Schwarz	10	*Acanthocinus pusillus* Kirby	64	*Chalcophora fortis* LeConte	1
Crypturgus borealis Swaine	11	*Acmaeops p. proteus* (Kirby)	5	*Chalcophora virginiensis* (Drury)	2
Crypturgus pusillus (Gyllenhal)	61	*Aegomorphus modestus* (Gyllenhal)	2	*Chrysobothris dentipes* (Germar)	1
Dendroctonus valens LeConte	261	*Anelaphus villosus* (F.)	14	*Chrysobothris femorata* (Oliver)	1
Dryocoetes autographus (Ratzeburg)	883	*Anoplodera pubera* (Say)	1	*Chrysobothris harrisi* Hentz	2
Dryocoetes betulae Hopkins	1	*Asemum australe* LeConte	3	*Chrysobothris sexsignata* Say	2
Gnathotrichus materiarius (Fitch)	242	*Asemum striatum* (L.)	701	*Chrysobothris trinervia* Kirby	1
Heteroborips seriatus (Blandford)	8	*Astylopsis collaris* (Haldeman)	2	*Dicerca divaricata* (Say)	7
Hylastes opacus Erichson	8	*Astylopsis macula* (Say)	1	*Dicerca tenebrosa* (Kirby)	2
Hylastes porculus Erichson	234	*Astylopsis sexguttata* (Say)	24	*Phaenops aeneola* (Melsheimer)	2
Hylurgops pinifex (Fitch)	39	*Bellamira scalaris* (Say)	1		
Hypothenemus californicus Hopkins	1	*Brachyleptura rubrica* (Say)	1		
Ips grandicollis (Eichhoff)	1372	*Clytus marginicollis* Laporte & Gory	1		
Ips pini (Say)	8	*Clytus ruricola* (Olivier)	5		
Lymantor decipiens (LeConte)	2	*Cyrtophorus verrucosus* (Olivier)	1		
Micracis suturalis LeConte	1	*Eutrichillus biguttatus* (LeConte)	4		
Monarthrum fasciatum (Say)	15	*Gaurotes cyanipennis* (Say)	1		
Monarthrum mali (Fitch)	19	*Graphisurus fasciatus* (Degeer)	33		
Orthotomicus caelatus (Eichhoff)	332	*Judolia cordifera* (Olivier)	5		
Pityogenes hopkinsi Swaine	38	*Microgoes oculatus* (LeConte)	1		
Pityokteines sparsus (LeConte)	1	*Monochamus notatus* (Drury)	123		
Pityophthorus cariniceps LeConte	2	*Monochamus scutellatus* (Say)	1592		
Pityophthorus consimilis LeConte	3	*Monochamus* spp. *(carolinensis* or *titillator)*	184		
Pityophthorus lautus Eichhoff	3	*Neoclytus acuminatus* (F.)	54		
Pityophthorus puberulus (LeConte)	112	*Orthosoma brunneum* (Forster)	2		
Polygraphus rufipennis (Kirby)	1	*Phymatodes aereus* (Newman)	8		

Pseudopityophthorus asperulus (LeConte)	10
Pseudopityophthorus minutissimus (Zimmermann)	11
Xyleborinus attenuatus (Blandford)	1
Xyleborinus saxesenii (Ratzeburg)	4
Xyleborus intrusus Blandford	1
Xyleborus xylographus (Say)	6
Xylosandrus germanus (Blandford)	26
Xyloterinus politus (Say)	1

Phymatodes testaceus (L.)	3
Prionus laticollis (Drury)	2
Rhagium i. inquisitor (L.)	8
Stictoleptura c. canadensis (Olivier)	1
Strangalepta abbreviata (Germar)	1
Strophiona nitens (Forster)	1
Tetropium cinnamopterum Kirby	2
Tetropium schwarzianum Casey	5
Trachysida mutabilis (Newman)	4
Trigonarthris subpubescens (Kirby)	1
Typocerus v. velutinus (Olivier)	1
Urgleptes signatus (LeConte)	1
Xylotrechus colonus (F.)	15
Xylotrechus integer (Haldeman)	2
Xylotrechus s. sagittatus (Germar)	36
